# Diagnostic Tumor Markers in Head and Neck Squamous Cell Carcinoma (HNSCC) in the Clinical Setting

**DOI:** 10.3389/fonc.2019.00827

**Published:** 2019-08-29

**Authors:** Panagiota Economopoulou, Remco de Bree, Ioannis Kotsantis, Amanda Psyrri

**Affiliations:** ^1^Section of Medical Oncology, Department of Internal Medicine, Faculty of Medicine, National and Kapodistrian University of Athens, Attikon University Hospital, Athens, Greece; ^2^Department of Head and Neck Surgical Oncology, UMC Utrecht Cancer Center, University Medical Center Utrecht, Utrecht, Netherlands

**Keywords:** head and neck cancer, HPV, tobacco, PET/CT scan, biomarkers, PD-L1, immunoscore

## Abstract

Head and neck squamous cell carcinoma (HNSCC) represents a group of tumors arising in the oral cavity, oropharynx, and larynx. Although HNSCC is traditionally associated with tobacco and alcohol consumption, a growing proportion of head and neck tumors, mainly of the oropharynx, are associated with Human Papilloma Virus (HPV). Recurrent/metastatic disease is characterized by dismal prognosis and there is an unmet need for the development of biomarkers for detection of early disease, accurate prediction of prognosis, and appropriate selection of therapy. Based on the REMARK guidelines, a variety of diagnostic and prognostic biomarkers are being evaluated in clinical trials but their clinical significance is doubtful. Herein, we will focus on biomarkers in HNSCC used in the clinical setting and we will illustrate their clinical relevance.

## Introduction

Head and neck squamous cell carcinoma (HNSCC) encompasses a heterogeneous group of malignancies that arise in the oral cavity, larynx and pharynx and are mainly associated with tobacco and alcohol consumption. In addition, epidemiological, molecular pathology and cell line data indicate that a substantial proportion of oropharyngeal cancers represents a sexually transmitted disease and is causally associated with high-risk human papillomaviruses (HPV), especially type 16 (1–3). HPV-associated oropharyngeal cancers (HPV-OSCCs) represent a distinct biological and clinical entity, have a distinct mutation landscape, and are characterized by markedly improved survival (4). The majority of HNSCC patients present with locoregionally advanced (LA) disease for which multimodality therapeutic approach is employed. Despite advances in diagnostics, treatment and surveillance, the 5-year progression-free survival (PFS) of HPV negative patients with LA disease is ~40–50% and survival rates for recurrent/metastatic (R/M) disease have not significantly changed over the past years.

Low survival rates associated with HNSCC are partly due to failure in early diagnosis. Indeed, only one third of HNSCC patients are diagnosed at an early stage (5); early diagnosis is mainly attributed to lack of appropriate screening and diagnostic biomarkers. Biomarkers are defined, according to the National Cancer Institute (NCI), as “a biological molecule found in blood, other body fluids, or tissues that is a sign of a normal or abnormal process, or of a condition or disease. A biomarker may be used to see how well the body responds to a treatment for a disease or condition” (6). Basically, biomarkers represent important tools that contribute to diagnosis, assess the likely course of the disease and predict response to treatment; thus, they are categorized as diagnostic, prognostic or predictive, respectively. Regarding HNSCC, although many biomarkers have been suggested to significantly impact diagnosis and prognosis, few of them have been validated for use in clinical practice. Indeed, a significant proportion of biomarkers in development are not introduced into clinical practice because they lack important features, such as high specificity and sensitivity, low cost, high positive predictive value, clinical relevance, and short turnaround time (7). The Transparent Reporting of a multivariable prediction model for Individual Prognosis or Diagnosis (TRIPOD) initiative, which has been developed with the joint effort of clinicians, statisticians, epidemiologists, and journal editors, has recommended a guideline for the reporting of studies developing, validating, or updating a prediction model, whether for diagnostic, or prognostic purposes (8). Based on the REMARK criteria (9), a handful of biomarkers are validated for clinical use in HNSCC. In this review, we will focus on established diagnostic biomarkers that are in clinical use in HNSCC, and we will discuss emerging biomarkers that are in development.

## Validated Biomarkers

### HPV

A growing proportion of oropharyngeal cancers is associated with HPV infection. More than 130 HPV types are known and classified as low-risk or high-risk based on their oncogenic potential; HPV16 is the most commonly found and is present in ~90% of HPV-OSCCs (10). Two meta-analyses of case-control studies have provided epidemiological evidence of the causative role of HPV in OSCC based on strong correlation between HPV16 exposure and HNSCC in certain anatomical sites (11, 12). Indeed, a strong correlation has been described between HPV-16 detection at the time of diagnosis with tonsillar cancer (odds ratios [OR], 15.1; 95% CI, 6.8–33.7) and OSCC (OR, 4.3; 95% CI, 2.1–8.9) (11). Biologically, the integration of high-risk HPV DNA into the host genome can lead to the expression of oncogenes E6 and E7 in the host cell; however, 60% of HPV-positive OSCC can contain extrachromosomal (episomal) virus. The E6 oncogene provokes the degradation of TP53. The E7 oncogene is implicated in binding and destabilizing of the tumor suppressor retinoblastoma (pRb) (3).

HPV-OSCC differs from HPV-driven cervical cancer, in which Pap smear and HPV DNA are widely used for screening in clinical practice; in HPV-OSCC there is no identified oropharyngeal premalignant lesion and the presence of HPV DNA in the oral cavity or oropharynx is not directly linked to subsequent development of HNSCC. Although detection of HPV16 DNA by Polymerase Chain Reaction (PCR) in both salivary oral rinses and plasma has demonstrated marked sensitivity and specificity (13), it has not been incorporated into clinical practice as a screening tool.

Detection of HPV DNA in saliva samples has been shown be a predictive tool for recurrence in HPV-associated OSCC (14–16). More specifically, in a study by Rettig et al. oral rinse samples were collected from patients with HPV-OSCC at diagnosis and at several timepoints after diagnosis and evaluated for HPV DNA. HPV DNA was detected in 54% of patients at diagnosis, but only in 5% of patients post-treatment. Importantly, all patients with HPV DNA positive samples post-treatment relapsed and persistent oral HPV infection correlated with disease free survival (DFS) and overall survival (OS) (14). Two additional smaller cohort studies have reported a correlation of HPV16 DNA detection in post-treatment oral rinses with survival (15, 16). These findings support the potential utility of HPV DNA detection in post-treatment oral rinses as a clinical test for the prediction of relapse.

In addition, large case control and prospective cohort studies have reported a strong correlation between seropositivity for antibodies against HPV16 oncogenic proteins E6/E7 and risk of OSCC. More specifically, Kreimer et al. evaluated pre-diagnostic plasma samples from patients with HNSCC and controls for antibodies against oncogenic proteins of HPV (17). These patients were participants in the European Prospective Investigation into Cancer and Nutrition (EPIC) study, which was conducted to assess the relationship between nutrition and cancer (18); samples were collected at a median of 6 years before diagnosis. Interestingly, HPV16 E6 seropositivity was found to be present in pre-diagnostic samples for 34.8% of patients with OSCC and 0.6% of controls (OR, 274; 95% CI, 110 to 681); most importantly, the increased risk of OSCC among HPV16 E6 seropositive participants was observed more than 10 years before diagnosis. Similarly, Agalliu et al. conducted a nested case-control study among 96,650 participants, who were cancer free at baseline, with available mouthwash samples in 2 prospective cohort studies: (1) the American Cancer Society Cancer Prevention Study II Nutrition Cohort and (2) the Prostate, Lung, Colorectal, and Ovarian Cancer Screening Trial. Among those participants, authors identified 132 cases of HNSCC during 3.9 years of follow up. It was shown that HPV-16 detection in the oral cavity, which preceded cancer diagnosis for an average of 3.9 years, was associated with a 22.4-fold increased risk of incident OSCC (95% CI, 1.8–276.7) after adjusting for smoking history and alcohol consumption, but not with risks of oral cavity or larynx SCC (19).

Furthermore, plasma and saliva HPV DNA have been shown to be important tools for predicting relapse in HPV-OSCC. In a recent retrospective study conducted in 93 patients with OSCC, among who 81 were HPV-positive, tumor DNA was detected in pre-treatment saliva and plasma samples in 53 and 67% of HPV-positive patients, respectively. When combined, pre-treatment saliva and plasma tumor DNA were 76% sensitive and 100% specific. Post-treatment saliva and plasma were 70% sensitive and 91% specific for disease recurrence (15). Finally, in a recent meta-analysis including 5 studies with both pre-treatment and post-treatment samples (*n* = 600 HNSCC patients), HPV DNA demonstrated a high pooled estimated specificity in detecting disease recurrence (100%) but an inferior pooled sensitivity (54%) (20). Recent technical advances in detecting circulating DNA using droplet digital PCR might improve sensitivity (21).

Therefore, HPV E6/E7 could be used as a clinical test to monitor treatment outcomes. Several studies have attempted to evaluate changes in HPV16 E6 and/or E7 antibody levels after treatment completion in patients with HPV-OSCC (22–28). The majority confirm the high incidence of seropositivity at diagnosis. Six out of 7 studies describe a decline in levels of HPV16 E6 antibodies post-treatment (22, 23, 25–27, 29). Among them, two showed a correlation between stable or increasing HPV16 E6 antibody levels and relapse (22, 25), one showed that patients who recurred had a lower clearance of antibody titers and three studies failed to demonstrate any significant association between post-treatment antibody levels and disease recurrence.

Compared to HNSCC unrelated to HPV, HPV-associated OSCC has emerged as a distinct disease entity with different clinical characteristics and a unique molecular profile, emphasizing the need for routine HPV testing of OSCC. Importantly, given the distinct clinical behavior and favorable prognosis of HPV-OSCC, a separate staging system has recently been developed for HPV-OSCC (30, 31). Indeed, the importance of HPV status as a diagnostic and prognostic biomarker necessitates the establishment of HPV testing and the incorporation of HPV status in therapeutic management; indeed, HPV positive and HPV negative OSCC are now being addressed separately in clinical trials. Nevertheless, there is currently no treatment de-intensification protocol recommended for HPV-OSCC and two recently published trials have shown reduced efficacy of anti-Epidermal Growth Factor (EGFR) monoclonal antibody cetuximab-based radiation compared to standard cisplatin chemoradiation (32, 33). More specifically, in the De-Escalate HPV trial, which was conducted in patients with low risk HPV-OSCC, cisplatin based chemoradiation was associated with survival benefit comared to cetuximab-radiotherapy combination, but this was a secondary endpoint and follow up was only 26 months (32). On the contrary, in the non-inferiority RTOG 1016 that did not focus on low risk HPV-OSCC, OS was a primary endpoint and it was found to be was higher in the cisplatin-radiotherapy arm after 5 years of follow up (33). Toxicity did not differ between arms in both studies. However, in the RTOG 1016 study several adverse events such as myelosuppression, anemia, nausea, vomiting, anorexia, dehydration, hyponatremia, kidney injury, and hearing impairment were significantly more frequent in the cisplatin group.

Both the College of American Pathologists and NCCN guidelines recommend HPV testing for all oropharyngeal tumors (34). In addition, The National Cancer Institute proposes the inclusion of HPV status as a risk stratification factor in current clinical trials addressing OSCC patients. However, it has been postulated that despite strong recommendations, HPV status is routinely assessed in 79% of OSCC cases in the UK and 67% of cases in the US, possibly due to costing issues and lack of predictive significance (35).

Of note, the role of HPV in HNSCC other than OSCC remains unclear. In carcinoma of the oral cavity, a report by Zafereo et al. indicated a high incidence of p16 overexpression (36.3%, especially in the tongue), but only 6% of oral cavity tumors were considered HPV-driven (36). In laryngeal cancer, the prevalence of HPV positivity is ~28% (37), but no correlation with survival has been reported (38). Therefore, HPV testing in patients with HNSCC other than OSCC is not routinely recommended outside of a clinical trial.

Detection strategies for HPV-OSCC differ in detection targets and include HPV DNA Polymerase Chain Reaction (PCR) for E6/E7 viral oncogenes, HPV E6/E7 mRNA detection quantitative reverse transcription-PCR (qRT-PCR), DNA *in situ* Hybridization (ISH), RNA ISH and p16 immunohistochemical staining (IHC) as a surrogate marker for HPV status (39). There is still no clear consensus about which method is the gold standard for HPV detection. For example, important advantages of standard PCR techniques include wide availability, high sensitivity (detection of HPV below one viral copy per genome cell) and cost effectiveness. However, PCR techniques are complex and have low specificity because they cannot distinguish between HPV that acts as an oncogenic driver and transcriptionally silent virus and have a high risk of contamination; these disadvantages hamper their capacity to detect a clinically relevant HPV infection (40). Importantly, detection of viral E6/E7 mRNA by RT-PCR is widely accepted as the gold standard for the detection of clinically significant HPV infection due to its generally high sensitivity, tumor-specific expression of the mRNA/DNA target and feasibility on formalin-fixed, paraffin-embedded tissue block (41). Significant limitations include that it is time-consuming and that its sensitivity decreases depending on quality of samples.

DNA ISH is a molecular method with high specificity, which enables direct detection of the presence of HPV virus in topographical relationship with pathological samples, ensuring that viral DNA originates from tumor cells and not surrounding tissues. ISH has the advantage of being feasible in both formalin-fixed and paraffin-embedded tissues, but it is a less sensitive method that is insufficiently clinically validated and is not currently used in routine screening (42). However, E6/E7-mRNA ISH, which allows direct visualization of viral transcripts in routinely processed formalin-fixed paraffin-embedded tissues, has sensitivity comparable to p16 IHC and qRT-PCR (43, 44).

P16INK4A (p16) is a tumor suppressor protein that regulates the cell cycle by inhibiting phosphorylation of CDK4 and CDK6, thus preventing Rb phosphorylation. During the HPV life cycle, the oncoprotein E7 inactivates the Rb protein, which results in the upregulation of various cell cycle associated proteins, including p16 (10). P16 is commonly used as a surrogate marker for HPV positivity and p16 IHC has been established as an essential complimentary procedure for HPV detection, due to its low cost, availability and high sensitivity (4, 45); however, low specificity limits its use as a standalone test (46). In addition, proper interpretation of p16 staining requires evaluation by trained pathologists and requires incorporation of histological and clinical information. Discordance rate between p16 IHC and direct detection of HPV DNA/RNA is estimated to be as high as 25%, with p16 + but HPV-tumors representing most of discordant cases (47).

As previously mentioned, detection of E6/E7 mRNA by PCR is suggested as the most enlightening method for determining HPV status. However, p16 IHC is widely used in clinical practice given its availability, simplicity and high sensitivity for detecting all high-risk types of HPV. Nevertheless, it cannot be utilized as a standalone test because of low specificity and a false positive rate where p16 expression is driven by non-viral mechanisms. Of note, outside the oropharynx, where the overall HPV infection rate is probably lower than 5%, p16 IHC is demonstrated to show very low or no correlation with HPV infection (48). In addition, there is substantial evidence that p16 positivity is associated with improved survival in R/M OSCC (49). Interestingly, tumors characterized as p16+/HPV-OSCCs have been correlated with poorer survival than p16+/HPV+ cancers (50). Because of its correlation with survival, p16-positivity is included in the recent World Health organization TNM classification for OPSCC.

In clinical practice, determination of HPV status usually starts with p16 IHC; subsequently, a different method of detection is used to reinforce reliability of the result. In a recent report by Fakhry et al. ASCO and CAP suggest p16 IHC as the initial test for HPV in tissue specimens. Additional HPV testing should be performed at the discretion of pathologist or treating physician (51). In a study by Weinberger et al. cases that were dually positive for p16 IHC and Real-time PCR HPV16 DNA were the biologically relevant HPV+ OSCC cases with favorable prognosis (4). A multimodality approach with p16 IHC followed by PCR or ISH on p16+ cases is proposed as the most appropriate to ensure high sensitivity and specificity. Indeed, Dutch and English groups have validated this approach reporting a sensitivity and specificity of almost 100% (52, 53).

### PET Imaging

18F-fluorodeoxyglucose PET (FDG-PET) is widely used as a diagnostic tool in HNSCC both for defining stage and evaluating treatment response. It has been shown to have higher sensitivity and a high negative predictive value compared to CT or MRI especially for identification of small lymph nodes of the neck (54, 55). This leads to a modification of treatment planning in approximately one third of the patients. Furthermore, in patients with cancer of unknown primary manifested as cervical lymph node metastases, FDG PET can identify the primary site in 25–38.5% of cases (56).

18-FDG pre-treatment parameters maximal and mean standardized uptake value (SUVmax and SUV mean), are most commonly used, despite flaws in calculation, and reproducibility (57). Several studies have shown that high pre-treatment SUV on PET/CT is an adverse prognostic factor in HNSC (58–61). In a metanalysis reported by Xie et al. both low pre-treatment and post-treatment SUV of the primary tumor was associated with improved disease-free survival (DFS), OS and local control (59); this result was confirmed in a subsequent meta-analysis by Zhang et al. (60). However, due to large differences in the SUV cut-off values and heterogeneity of various studies, the clinical utility of the results of these metanalyses is questioned.

Post-treatment FDG-PET is also commonly used for HNC-response assessment after definitive radiotherapy or chemoradiotherapy. A meta-analysis of 51 studies that included 2,335 patients showed that the sensitivity, specificity, positive predictive value (PPV), and negative predictive value (NPV) of FDG PET for the detection of residual primary HNSCC were 94, 82, 75, and 95%, respectively, (55). Notably, a positive FDG PET/CT study in the post-treatment evaluation needs thorough consideration of clinical information and endoscopy findings and might require biopsy of suspicious positive sites to confirm diagnosis, as radiation-induced inflammation can also lead to FDG uptake (62). Ong et al. retrospectively evaluated the records of patients with HNSCC treated with concurrent CRT who have underwent a PET/CT 8 or more weeks after treatment. PET/CT findings were confirmed by biopsy, neck dissection or imaging follow up. In the NPV and specificity of FDG PET/CT for residual nodal disease were 97 and 89%, respectively, whereas sensitivity was 71% and PPV was 38%. Specificity and NPV of PET/CT increased in the subgroup of patients without residual enlarged neck nodes at CT (63). On the contrary, in a study reported by Waldron et al. that included 339 patients with N2/N3, both NPV and sensitivity of PET/CT were low (53 and 73% respectively) (64). Interestingly, PET/CT scan has been reported to have high NPV and sensitivity in HPV-related HNSCC (65–67).

Mehanna et al. conducted a prospective randomized phase III trial to assess the utility of PET/CT as a biomarker of residual disease and as a tool to avoid unnecessary neck dissection post radical chemoradiotherapy (CRT) in patients with advanced N2/N3 disease. In this non-inferiority study, patients were randomized to either surveillance via PET/CT, which was performed 12 weeks post CRT, and neck dissection in the case of incomplete response or equivocal findings or planned neck dissection. Overall survival was found to be similar among the two groups, but PET/CT-guided surveillance was more cost effective and resulted in fewer surgical operations (68).

Importantly, positive PET/CT findings might be more properly interpreted if time interval between treatment completion and PET/CT exceeds 12 weeks. The results of the ECLYPS study, which sought to implement PET/CT findings according to international guidelines in patients with LA HNSCC treated with radical CRT, suggested that FDG-PET/CT can successfully identify residual neck disease 12 weeks after CRT. Importantly, although its sensitivity was high in detecting residual disease in patients who relapsed up to a 9 month horizon after imaging, sensitivity was lower for disease manifesting up to 12 months after imaging (sensitivity, 59.7%) (69). In addition, even with optimal timing, SUV cutoff is not a reliable biomarker for discrimination between cancer and radiation-induced inflammation (44); however, a reduction in SUVmax of >50% has been associated with improved outcomes (70).

### Tobacco

Although smoking rates continue to decrease across the United States (US), they are particularly high among cancer patients. Indeed, ~60% of newly diagnosed cancer patients are characterized as current or former smokers, with the highest numbers in lung cancer and HNSCC (71). The odds ratios of developing HNSCC is 2.37 (1.66–3.39) for tobacco only users, but combined alcohol and smoking consumption has a more multiplicative effect, with an odds ratio of 5.73 (3.62–9.06) (72). Smoking rates at diagnosis in patients with HNSCC range from 26.4 to 56% (73, 74) and vary across subsites, with the highest rates observed in laryngeal cancer (73, 75).

Most importantly, tobacco consumption in HNSCC is associated with inferior treatment-related outcomes, including surgical outcomes, and radiation efficacy. Furthermore, smoking at diagnosis is associated with reduced survival rates, higher risk for second primary cancers such of the lung and esophagus, increased risk of comorbidities and competing causes of death (76, 77). In a landmark study, Ang et al. conducted a retrospective analysis in patients with stage III-IV OSCC who participated in the RTOG 0129 study that compared accelerated-fractionation radiotherapy with standard-fractionation radiotherapy, each combined with cisplatin therapy. The authors sought to assess the prognostic significance of HPV status and association with survival. Using recursive partitioning analysis, they incorporated tobacco consumption into a classification model that was based on four factors: HPV status, pack-years of tobacco smoking, tumor stage, and nodal stage; a cutoff of 10 pack years of smoking was reported to be the best predictor of survival. Patients were classified as having a low, intermediate or high risk of death (78). This study demonstrated that although HPV status is a strong predictor of survival, the favorable biologic behavior of an HPV-positive tumor may be altered by tobacco exposure; tobacco-driven molecular alterations may decrease effectiveness of radiation treatment.

A year later Gillison et al. retrospectively evaluated patients with OSCC enrolled in the aforementioned RTOG 0129 trial and the RTOG 2003 for HPV status and tobacco consumption. After adjustment for p16 and other factors, it was demonstrated that risk of death increased by 1% per pack-year or 2% per year of smoking in both trials. In addition, in RTOG 9003, overall survival was significantly associated with tobacco exposure during radiation treatment (79). Similar results had been previously reported in patients with HNSCC early in 1993; Browman et al. had demonstrated that response rate and survival were decreased among patients who smoked during radiotherapy (80). In addition, Chen et al. conducted a matched control study in patients with HNSCC receiving radiotherapy and demonstrated reduced treatment-outcomes in patients who continued to smoke as opposed to matched smokers who have quit (81). Suggested mechanisms for smoking-induced effects on survival in HNSCC include inflammation and tobacco carcinogen-induced DNA damage (82).

Although smoking status has been shown to profoundly affect treatment outcomes in HPV-related OSCC, it has not been incorporated into the novel staging system specifically developed for HPV-OSCC in the American Joint Committee on Cancer Staging (AJCC) 8th edition. Historically, successful stage grouping yields similar survival rates for patients among the same T and N subgroup and significantly different survival rates across subgroups; in addition, patients must be equally distributed between groups (83). In an attempt to provide improved predictive ability that complies with the distinct outcomes expected for patients who suffer from HPV-associated as compared to HPV-negative OSCC, Dahlstrom et al. developed a proposal for a new staging grouping for HPV-related OSCC based on recursive partitioning analysis (RPA). Indeed, stratification of patients based on smoking history using the cutoff of 10 pack years proposed by Ang et al. revealed a different PFS impact based on smoking status. However, when these groups were compared within each stage group, no difference in survival was found (84). Therefore, the authors concluded that although smoking is an important prognostic factor for HPV-OSCC, there is no need to include it into the new staging system, if TNM stage accurately reflects prognosis. Nevertheless, as the new AJCC 8th edition TNM classification starts to be used in clinical practice, new data will be encompassed into cancer registries, and these may urge future reclassification of prognostic stage groupings that might include smoking as a classification factor.

### Immunoscore

Although TNM is a good prognostic system that accurately reflects patient prognosis, clinical outcomes of patients distributed across TNM stages might frequently be different than expected. For instance, some patients with small tumor burden recur quickly, whereas others with metastatic disease have an unexpectedly favorable prognosis. In recent years, it has been well-established that the immune system plays a pivotal role in the control of tumor growth (85) and it has been suggested that potentially invading cancer cells are held in an equilibrium state that is controlled by the immune system (86). Subsequently, certain tumors escape and become clinically apparent.

Accumulating evidence has emphasized the need for the development of immunological biomarkers that can offer prognostic information and facilitate clinical decision-making. Tumor-infiltrating immune cells, including T and B lymphocytes, macrophages or neutrophils can have either a negative or a positive effect on tumor expansion. Evaluation of the dynamics and functional roles of different subsets of tumor infiltrating cells in the tumor suppressive microenvironment could improve our knowledge of immunology and define subgroups of patients that are more likely to respond to immunotherapy. Cytotoxic CD8+ tumor infiltrating lymphocytes (TILs) are thought to be the major effector immune cells directed against tumor cells and have been shown to have prognostic significance in many solid tumors (87–89). On the other hand, regulatory T cells (Tregs) inhibit immune response and counteract cytotoxic T cells. Inconsistent results have been reported regarding prognostic significance of Tregs, with several studies showing association with poor prognosis in a variety of malignancies including breast, lung, cervical and ovarian cancers, while others demonstrating favorable prognostic significance, e.g., in colorectal cancer (90).

HNSCC is a disease characterized by profound immunosuppression (86). Several studies have reported a significantly increased density of TILS in HPV-positive as compared to in HPV-negative OSCC, which implies a more potent anti-tumoral immune response in HPV-OSCC (91–93). This has been suggested as the mechanism for improved outcome in HPV-OSCC across studies. In addition, high levels of TILs have been associated with improved survival in HPV-OSCC (94, 95). Interestingly, patients with HPV-positive disease and low TIL levels did not show a survival advantage compared with HPV-negative counterparts (94). On the contrary, HPV-positive patients with high TILs have been shown to have superior survival (95), suggesting the use of TILs as a future biomarker for de-intensification treatment patient selection in HPV-positive disease. High TILs have also been associated with improved survival in tobacco-related HNSCC (96, 97).

Furthermore, levels of both CD8+ and CD3+ T cells have been associated with increased overall survival after definitive chemoradiotherapy, both in HPV-positive, and HPV-negative HNSCC (98, 99). In a more recent multicenter study of patients with HNSCC after post-operative chemoradiotherapy, high CD8 TILs density measured on tumor periphery, tumor stroma, and tumor cell area was predictive for improved OS (98). Interestingly, in another study, only stroma TILs infiltration was associated with increased survival (100).

Of note, HPV-OSCC has been shown in several studies to possess a high degree of T reg infiltration (95, 101, 102). Tregs have been shown to correlate with favorable OS and locoregional control (95, 102), possibly reflecting the downregulation of inflammation which triggers the initiation of carcinogenesis (103).

A clinical application of the prognostic significance of TILs is the establishment of an Immunoscore, which emerges as a potential algorithm to define antitumor immune responses using quantitative pathology. Immunoscore is based on the quantification of CD3+ and CD8+ TILs in the tumor core and the invasive margin of resected tumors and uses this numeration of TILs to provide a score ranging from Immunoscore 0, when low numbers of both cell types are described in both regions, to Immunoscore 4, when high numbers are described in both regions. Immunoscore has been applied in colorectal cancer in large cohorts (104). In HNSCC, both CD8+ T cells infiltrate in the tumor component of the invasive margin and PD-L1 expression in the tumor were predictive of disease recurrence (105).

### PD-L1

Immune checkpoints modulate signaling and either inhibit or enhance T-cell response. Cytotoxic T lymphocyte Antigen 4 (CTLA-4) and Programmed Cell Death protein 1 (PD-1) are distinct examples of co-inhibitory molecules; because PD-L1 mediates the inhibition of T cell activity, it can be theoretically assumed that high expression might result in poor survival. PD-L1 is upregulated under inflammatory conditions and is expressed in T-cell enriched tumors (106). In a laryngeal HNSCC cohort, high PD-L1 expression assessed by Automated Quantitative protein Analysis (AQUA) was found to positively correlate with disease outcome (96). In a recent report by Yang et al. PD-L1 was shown to correlate with improved PFS but not OS in patients with advanced HNSCC. Interestingly, patients with combined low expression of TILs and high expression of PD-L1 were characterized by dismal survival (107). Another retrospective analysis that assessed PD-L1 expression in a large cohort of patients, demonstrated that high PD-L1 expression was the strongest predictor of worse outcome, independent of tumor origin (108). In cancers of the oral cavity, increased PD-L1 expression has been also shown to correlate with poor survival (109).

In early immunotherapy studies, PD-L1 expression was shown to be associated with the rate of response to immune checkpoint inhibitors and was therefore established as the most commonly used predictive biomarker (110, 111). Therefore, evaluation of PD-L1 expression currently represents a reference biomarker for clinical trials. However, accurate measurement of PD-L1 protein levels in FFPE tumor samples is hampered by technical issues, such as the use of different assays and antibodies across different studies and tumor types, the variability of cut-off values, and scoring methods and the lack of standardized methods (112). In addition, intertumoral, and intratumoral heterogeneity hampers homogeneous PD-L1 evaluation.

Indeed, it is clear that PD-L1 is an imperfect albeit useful predictive biomarker. In addition, no other biomarker has shown correlation with immunotherapy response in HNSCC. In the phase III Keynote-040 study, which assessed the efficacy of pembrolizumab vs. investigator's choice (methotrexate, docetaxel, or cetuximab) in platinum resistant R/M HNSCC, a statistically significant difference in OS in favor of pembrolizumab was shown in patients with combined positive score (CPS, defined as ≥1 of expression in both tumor and mononuclear inflammatory cells) ≥1 (8.7 months vs. 7.1 months, *p* = 0.0078), and in patients with CPS ≥50 (11.6 vs. 7.9 months, *p* = 0.0017) (113).

Importantly, the results of the phase III Keynote 048 trial, which compared pembrolizumab alone or in combination with chemotherapy vs. EXTREME in treatment-naïve HNSCC, were recently presented (114). Pembrolizumab significantly improved OS over EXTREME in the PD-L1 CPS ≥ 20 (14.9 vs. 10.7 months; *p* = 0.0007) and ≥1 (12.3 vs. 10.3 months, *p* = 0.0086) subgroups; and was non-inferior in the total population (11.5 vs. 10.7 months *p* = 0.0199) with favorable safety. Furthermore, pembrolizumab and chemotherapy combination was superior to EXTREME in terms of OS in both the CPS ≥20 (14.7 vs. 11.0 months, *p* = 0.0004) and CPS ≥1 (13.6 vs. 10.4 months, *p* < 0.0001) populations and in the total population (13.0 vs. 10.7 months, *p* = 0.0034). Based on these results, in June 2019, FDA has approved pembrolizumab for the first line treatment of patients with recurrent/metastatic HNSCC. Pembrolizumab and chemotherapy combination has been approved for all patients, while single agent pembrolizumab has been approved for patients with PD-L1 CPS>1; therefore, assessment of PD-L1 score has become clinically relevant for treatment selection.

In addition to pembrolizumab, other PD-1/PD-L1 antibodies have been investigated in HNSCC. Nivolumab has been assessed in the landmark phase III CHECKMATE 141 trial, which compared nivolumab to 2nd line chemotherapy or cetuximab in patients with platinum refractory HNSCC (115). Patients treated with Nivolumab had a significant improvement in OS; although OS benefit was not statistically significant in the subgroup of patients with a PD-L1 expression <1%, nivolumab received FDA approval for the treatment of platinum refractory disease regardless PD-L1 status. On the other hand, the anti-PD-L1 antibody durvalumab has been evaluated in a phase II study in 111 patients with platinum pre-treated HNSCC; a high PD-L1 expression level of ≥ 25% was required for inclusion in the study (116). Durvalumab was associated with an ORR of 16.2%; interestingly, HPV-positive patients had a numerically higher response rate than HPV-negative patients (29.4 vs. 10.9%).

## Emerging Biomarkers

### Skeletal Muscle Mass

In recent years, body composition research in cancer patients has accelerated due to the use of routinely performed, diagnostic CT or MRI imaging for quantification of the different body compartments. Evidence is mounting that an abnormal body composition, in specific a low skeletal muscle mass (SMM), is an adverse predictive and prognostic factor in cancer patients. The most commonly used method for SMM measurement in cancer patients is on CT imaging a single CT slide at the level of the third lumbar vertebra (L3). The cross-sectional muscle area at this level is then normalized for height by dividing it by the squared height, in order to calculate the lumbar skeletal muscle index (lumbar SMI). This method has been validated in studies using whole body MRI, in which it has been shown that skeletal muscle area on a single transversal slice at the level of L3 is strongly correlated with total skeletal muscle volume as measured using whole body MRI (117, 118).

Abdominal CT imaging is frequently routinely performed in patients with certain cancer types during diagnostic work-up and follow-up, allowing for routine evaluation of SMM in these patients without the burden or costs of additional diagnostics. However, because abdominal CT imaging is not routinely performed in head and neck cancer (HNC), this method is not clinically applicable in HNC patients. It is known that risk factors for having a low SMM, such as malnutrition, and chronic inflammation, are highly prevalent in HNC patients (119).

Recently, a novel method to assess SMM on a single transversal CT slice at the level of the third cervical vertebra (C3) was published (120). Using this method, skeletal muscle mass is assessed measuring the skeletal muscle areas of the paravertebral muscles and the sternocleidomastoid muscles at the level of the C3 vertebra. This method allows for evaluation of SMM in HNC patients on routinely performed imaging, in a similar manner as is used in patients with other types of cancer. This measurement method has been validated in studies using whole body MRI (121), appears to be also applicable on MRI of the head and neck (122) and has a high interobserver and intraobserver agreement (123, 124). Also others found measurement of skeletal muscle area at level C3 a good alternative for measurement at level L3 (125).

Low SMM has been found to be predictive for complications and toxicity and prognostic for survival in HNC patients. Low SMM was predictive for wound complications, in particular pharyngocutaneous fistula after total laryngectomy in several studies. In 235 HNSCC patients undergoing total laryngectomy patients with low SMM (measured at C3) had more pharyngocutaneous fistulas than patients with normal SMM (34.9 vs. 20.6%, *p* = 0.019) and prolonged hospital stay (median 17 vs. 14 days, *p* < 0.001). In multivariate analysis, low SMM (HR: 2.1 95% CI: 1.5–2.9), and high N-stage were significant prognosticators of decreased overall survival (126). In retrospective analysis of 60 advanced laryngeal cancer patients who underwent total laryngectomy low skeletal mass area of paravertebral muscles at level C3 was predictive for wound complications (127). In a retrospective medical chart review of 70 patients who underwent laryngectomy low SMM, as measured at the level of L3, was an independent predictor of the occurrence of (wound) complications (128).

Low SMM was found to be also predictive for chemotherapy dose-limiting toxicity (CLDT) in patients with locally advanced HNC treated with 3 weekly high dose cisplatin concurrent radiotherapy using the C3 measurement tool. Patients with low SMM experienced CDLT three times more frequently than patients with normal SMM (44.3 vs. 13.7%, *p* < 0.001) and received a higher dose of chemotherapy/kg lean body mass (estimated from SMM, *p* = 0.044). At multivariate analysis, low SMM was independently inversely associated with CDLT (OR 0.93, 95%CI: 0.88–0.98). Patients experiencing CDLT had a lower overall survival than patients who did not (mean 36.6 vs. 54.2 months, *p* = 0.038) (129). In a study of 246 HNC patients with low SMM (measured at level C3) receiving concurrent chemoradiation were more likely to require radiation treatment breaks and suffer chemotherapy toxicity. Low SMM was also associated with worse overall survival and progression-free survival in HNC patients, except for p16-positive oropharyngeal cancer patients (130). Also in another study of 221 HNC patients receiving concurrent chemoradiation, patients with low SMM (measured at L3) required radiotherapy interruption more frequently (131).

In a retrospective study of 441 HNC patients low SMM (measured at L3) was associated with significantly poorer survival compared to non-sarcopenic patients, with the strongest association seen among overweight/obese patients (132). This negative impact on overall survival was confirmed in another study of 260 HNC patients in which SMM was measured at L3 (133). Another recent study showed that pre-treatment and post-treatment diminished SMM measured at L3 had about 3-fold increased risk of overall recurrence or death (134). Low SMM measured at L3 was also a prognostic factor affecting overall survival in advanced oropharyngeal cancer patients, independent of HPV status (135).

The exact mechanisms of the relation between low SMM and adverse outcomes are currently unknown. It is also unknown to which extent the negative effect of sarcopenia can be overturned by improving a patient's physical condition and nutritional status before and during treatment. Treatment strategies may be personalized to the patient's specific body composition to decrease the risk of adverse outcomes.

### Next Generation Sequencing (NGS)

During the past few years, next generation sequencing (NGS) offers the opportunity for molecular characterization and has therefore expaned our knowledge of genetic profiles in a variety of solid tumors. In head and neck cancer, several retrospective studies have reported the presence of mutations of genes in cohorts of largely HPV-negative HNSCC, most notably TP53, PIK3CA, CDKN2A, the TERT promoter, and NOTCH pathway gene alterations (136–139). In a landmark report, Stransky et al. analyzed whole-exome sequencing data in 74 HNSCC tumor-normal pairs and found that the majority harbored mutations associated with tobacco exposure (136). In addition to identifying previously known HNSCC genes (TP53, CDKN2A, PTEN, PIK3CA, and HRAS), the authors also demonstrated mutations in genes that regulate squamous differentiation (e.g., NOTCH1, IRF6, and TP63) (136).

On the other hand, Seiwert et al. focused more on HPV positive HNSCC by performing massively parallel sequencing of 617 cancer-associated genes in 120 matched HNSCC tumor/normal samples of which 42.5% were HPV-positive (140). It was demonstrated that HPV-positive tumors have a significantly different mutational profile compared to their HPV-negative counterparts, with unique mutations in DDX3X, FGFR2/3 genes and aberrations in PIK3CA, KRAS, MLL2/3, and NOTCH1 (Seiwert). In a more recently published prospective study, target sequencing was performed in 92 HNSCC tumors and matched blood samples (141). The most common mutations identified were TP53 was (51%), CDKN2A (25%), CCND1 (24%), and PIK3CA (21%); TP53, CDKN2A, and CCND1 gene alterations were present more frequently in HPV-negative tumors, while HPV-positive tumors were significantly associated with immune signature-related genes. In addition, several mutations such as NOTCH1 CDKN2A and TP53 were found to be prognostic for poor survival (141). Table 1 summarizes the role of biomarkers in HNSCC.

**Table 1:** Role of biomarkers in HNSCC.

**Marker**	**Mechanism**	**Prognostic role**	**Predictive role**	**Diagnostic role**	**Limitations**	**Validated**
HPV	Oncogenesis-driver in OSCC	Yes	No (2 clinical trials negative, other trials still ongoing)	Yes (Cancer of unknown primary presenting with cervical LNs)	Lack of specificity, applicable only in OSCC	Yes
PET imaging	-	Yes (high pretreatment SUV)	Yes (indication of residual disease for performing LN dissection)	Yes (stage, treatment response)	Appropriate interval between treatment completion and PET unclear, not always available	Yes
Tobacco	Inflammation and tobacco carcinogen-induced DNA damage	Yes (inferior treatment outcomes)	No	No	Demographic parameter	Yes
Immunoscore	Quantification of CD3+ and CD8+ TILs in the tumor core and the invasive margin of resected tumors	Yes (high number of TILs improve survival)	No (being assessed for response to immunotherapy)	No	Not always available	Yes
PD-L1	Mediates the inhibition of T cell activity	Yes (conflicting)	Yes (response to immunotherapy)	No	Technical issues in measurement	Yes
Skeletal muscle mass	Abnormal body composition	Yes (poor survival)	Yes (wound complication, fistula after laryngectomy, chemotherapy toxicity)	No		No
Next generation sequencing	Oncogenesis drivers	Yes (TP53, NOTCH1, CDKN2A mutations)	No	No	Cost, Not always avalaible	No

*HPV, Human Papilloma Virus; LN, lymphnode; OSCC, Oropharyngeal Squamous Cell Carcinoma; PD-L1, Programmed Death-Ligand-1; SUV, Standardized Uptake Value; TILs, Tumor Infiltrating Lymphocytes*.

## Conclusions

Identification of appropriate biomarkers can lead to early detection of HNSCC. It is commonly accepted that a tumor biomarker is a molecular signal or process-based change that reflects the status of an underlying malignant disease and can be detected by one or more assays or tests. However, a tumor biomarker must be characterized by accuracy, reproducibility and reliability in order to be clinically useful and guide management. In HNSCC, several biomarkers have emerged, showing promising results in diagnosis, early detection and prognosis of HNSCC. HPV DNA/p16 for the determination of HPV status, PET imaging and PDL1 are validated diagnostic and prognostic/predictive biomarkers currently used in clinical practice. In the future, other patterns of molecular markers, such as interferon-γ signature and tumor mutational burden, alone or in co-ordination with imaging markers, could be utilized for early detection and prognosis of HNSCC. Importantly, better understanding of the complex tumor-immune cell interactions will contribute to the development of exceptional prognostic markers and therapeutic avenues, with the view to improve patient outcomes.

## Author Contributions

PE: writing-original draft preparation. RdB: editing and writing of the manuscript. IK: data acquisition and interpretation. AP: conceptualization, design, writing-review and editing, and supervision. All authors had read and approved the final manuscript.

### Conflict of Interest Statement

The authors declare that the research was conducted in the absence of any commercial or financial relationships that could be construed as a potential conflict of interest.
